# Long Lasting Microvascular Tone Alteration in Rat Offspring Exposed *In Utero* to Maternal Hyperglycaemia

**DOI:** 10.1371/journal.pone.0146830

**Published:** 2016-01-12

**Authors:** Emilie Vessières, Abdallah Dib, Jennifer Bourreau, Eric Lelièvre, Marc-Antoine Custaud, Martine Lelièvre-Pégorier, Laurent Loufrani, Daniel Henrion, Céline Fassot

**Affiliations:** 1 CNRS UMR 6214, INSERM U1083, University of Angers, Angers, France; 2 Cardiovascular Functions In Vitro (CARFI) platform, Angers, France; 3 University Hospital (CHU) of Angers, Angers, France; 4 INSERM U872, Cordeliers Research Center, University of Pierre and Marie Curie, and University of Paris-Descartes, Paris, France; INSERM UMRS 1138, FRANCE

## Abstract

Epidemiologic studies have demonstrated that cardiovascular risk is not only determined by conventional risk factors in adulthood, but also by early life events which may reprogram vascular function. To evaluate the effect of maternal diabetes on fetal programming of vascular tone in offspring and its evolution during adulthood, we investigated vascular reactivity of third order mesenteric arteries from diabetic mother offspring (DMO) and control mother offspring (CMO) aged 3 and 18 months. In arteries isolated from DMO the relaxation induced by prostacyclin analogues was reduced in both 3- and 18-month old animals although endothelium (acetylcholine)-mediated relaxation was reduced in 18-month old DMO only. Endothelium-independent (sodium nitroprusside) relaxation was not affected. Pressure-induced myogenic tone, which controls local blood flow, was reduced in 18-month old CMO compared to 3-month old CMO. Interestingly, myogenic tone was maintained at a high level in 18-month old DMO even though agonist-induced vasoconstriction was not altered. These perturbations, in 18-months old DMO rats, were associated with an increased pMLC/MLC, pPKA/PKA ratio and an activated RhoA protein. Thus, we highlighted perturbations in the reactivity of resistance mesenteric arteries in DMO, at as early as 3 months of age, followed by the maintenance of high myogenic tone in older rats. These modifications are in favour of excessive vasoconstrictor tone. These results evidenced a fetal programming of vascular functions of resistance arteries in adult rats exposed *in utero* to maternal diabetes, which could explain a re-setting of vascular functions and, at least in part, the occurrence of hypertension later in life.

## Introduction

The concept of “fetal programming” is defined as the process whereby an adverse *in utero* environmental stimulus during the critical period of development can induce cardiovascular diseases during adulthood [1]. Epidemiological studies have clearly demonstrated that changes in the intra-uterine environment during specific windows of fetal development are significant causes of fetal stress [2,3], leading to loss of structure/function and pre-emptive adaptations to an adverse post-natal environment [4]. The mechanisms by which these alterations occur are not fully understood but they can include: 1) permanent structural changes in organs; 2) epigenetic modifications and 3) permanent effects on the regulation of cellular ageing [5].

Environmental perturbations during pregnancy predispose offspring to altered vascular function, leading to hypertension [6,7,8,9] and cardiovascular disease later in life [10]. It is well known that the vascular tone of small arteries (i.e. mesenteric arteries) is the main determinant of arterial resistance and ultimately of arterial pressure. Vascular tone is defined as blood vessel response by vasodilation or vasoconstriction to external stimuli in order to regulate transmural pressure [11]. In resistance arteries, endothelium-dependent vasodilation, mediated by a combination of several vasoactive substances including nitric oxide (NO) and prostacyclin (PGI_2_), acts directly to reduce vascular tone [12,13]. Conversely, myogenic tone (MT) is a contraction of small resistance arteries in response to increased intraluminal pressure [11]. This response is independent of neural, metabolic, and hormonal influences but directly implicates smooth muscle cells with dynamic remodelling of actin cytoskeleton and actin polymerization [14]. In addition, myosin light chain (MLC) phosphorylation, filamin and profilin are key regulators of the dynamic reorganization of actin filaments [15,16]. Thus equilibrium between MT and dilation allows efficient control of local blood flow and arterial pressure through its effect on vascular calibre in peripheral tissues. Studies using models of animals exposed *in utero* to maternal diabetes or obesity have shown the occurrence of endothelial dysfunction in large and resistance arteries [17,18,19]. These vascular perturbations have only been linked to alterations in the NO pathway [20,21,22,23]. However, in an experimental model of *in utero* exposure to moderate maternal hyperglycaemia, our group has demonstrated that, prior to hypertension development [24], the aortae of offspring (DMO, 3 months old) of young diabetic mothers, showed a specific alteration of PGI_2_-induced vasodilation linked to a functional reduction in prostacyclin receptor (IP receptor) activity [25]. As studies seem to focus on unbalanced vascular tone due to vasodilation perturbations, little is known about vasoconstrictor pathways involved in the control of vascular tone.

In this study, we aimed to determine whether maternal hyperglycaemia during pregnancy could affect resistance artery tone in offspring at a pre-hypertensive stage and later in life. We hypothesize that fetal programming of vascular tone could impact the regulation of arterial pressure in DMO through an impaired balance between vasodilator and vasoconstrictor responses.

## Research Design and Methods

### Animals

Pregnant Sprague–Dawley rats, weighing 250-300g, were made diabetic on day 0 of gestation by a single intraperitoneal injection of streptozotocin (Sigma, 35mg/kg), as previously described [25]. The diabetic state was checked in fasted rats by measuring the plasma glucose concentration (AccuChek®, Roche) in their tail blood. Only pregnant females whose plasma glucose ranged between 15 and 25mmol/l were included in the study. This diabetic status was confirmed every two days until delivery. On the day of delivery, the newborn rats were weighed. Each litter was then reduced to 10 pups. All animals were kept in a temperature and light controlled room, at 21°C with a 12 hour light cycle. They had ad libitum access to food (SDS Laboratory) and tap water. Eight to fourteen different litters of DMO and CMO were used for the entire study. After weaning, males from each litter were split into 4 different groups: 2 groups for blood pressure measurements and 2 groups for vascular tone measurements. For each type of measurement, there was one group of 3-month old and one group of 18-month old rats. Each experiment was performed on animals from at least 3 different litters, (taking into account that the same litters are represented in each group of experiments). All experiments were conducted in accordance with the institutional guidelines and the recommendations for the care and use of laboratory animals as put forward by the French Ministry of Agriculture. The protocol was approved by the Ethic Committee of “Pays de Loire” (permit # CEEA.2011.42).

### Arterial blood pressure measurements

Blood pressure (BP) was measured in conscious CMO and DMO at 3 and 18 months of age as previously described [25]. In short, animals were anesthetized using sodium pentobarbital anaesthesia (60mg/kg, i.e.; Sanofi) and an arterial catheter (PE-50 fused to PE-10: internal diameter of 0.28mm, and external diameter of 0.61mm) was inserted into the femoral artery. Upon regaining consciousness, animals were housed in individual cages. After 3 days of recovery, BP was recorded in conscious unrestrained animals over a period of 30 minutes. The arterial catheter was connected to a pressure transducer (P10EZ, Becton Dickinson), linked with a Gould RS 3400 polygraph in order to continuously measure pulsatile BP.

### Vascular function of resistance arteries

Vascular function was assessed in isolated mesenteric arteries of 3 and 18-month old rats.

#### Pharmacological study

contraction and vasodilation of isolated mesenteric arteries was assessed in response to KCl (80mM) and cumulative dose-response curves of phenylephrine (PE), acetylcholine (Ach, endothelium-dependent vasodilator), sodium nitroprusside (SNP, endothelium independent vasodilator) and beraprost (PGI_2_ analogue, endothelium-derived vasodilator). Reagents were purchased from Sigma-Aldrich (PE, Ach and SNP) and Cayman (beraprost). Arterial segments (2mm long) were dissected and mounted on a wire-myograph (Danish MyoTechnology, DMT) as previously described [26]. Briefly, 2 tungsten wires (25μm diameter) were inserted into the lumen of the artery and fixed respectively to a force transducer and a micrometer. Arteries were bathed in a 5ml organ bath containing a physiological salt solution (PSS) maintained at a pH of 7.4, a pO_2_ of 160mmHg and a pCO_2_ of 37mmHg. After wall tension normalization, arteries were allowed to stabilize for 30 minutes. Endothelial integrity was assessed after pre-constriction with 10^-6^M PE by evaluating the vasodilator effect of 10^−6^ M Ach. Endothelium-independent relaxation to SNP was obtained at the end of the protocol. Cumulative concentration-response curve to Ach, SNP and beraprost were performed after PE-induced pre-constriction (10^-6^M). Data were expressed as a % relaxation of PE-induced pre-contraction.

#### Study of pressure-dependent tone

mesenteric arteries were cannulated at both ends in a video-monitored perfusion system (LSI) as previously described [27]. Briefly, third order arteries were bathed in a physiological salt solution (pH 7.4, PO_2_ 160mmHg, PCO_2_ 37mmHg). Pressure was controlled by a servo-perfusion system. Pressure was increased little by little from 10 to 125mmHg without intraluminal flow [28]. At the end of each experiment, arterial segments were superfused with a Ca^2+^-free physiological salt solution containing ethylenbis-(oxyethylenenitrolo) tetra-acetic acid (EGTA; 2mM), papaverin (10^-4^M) and SNP (10^-5^M); then pressure steps were repeated to determine the passive diameter of the arteries [29]. Results are given in micrometres for artery diameter. Myogenic tone was expressed as the percentage of passive diameter (measured diameter/passive diameter×100).

### Histomorphometry analysis

Morphological studies were performed on mesenteric arteries fixed in 4% buffered formaldehyde and embedded in OCT. Sections (7μm) were stained with orcein for elastic fibre detection in order to measure histomorphometric parameters. Internal and external media perimeters, intima-media thickness and medial cross-sectional area were determined after image acquisition (Nikon Eclipse E600 microscope, Sony camera), and analysed using ImageJ software (NIH).

### Blood parameters

Before sacrifice, glycaemia was quantified in fed animals, on a sample of arterial blood with a glucometer. Serum blood level of 6-keto-PGF1α, a stable metabolite of PGI_2_, was measured in CMO and DMO, using a commercially available kit (Cayman).

### Western blot analysis of protein expression

Western blot analysis of proteins of interest was performed following previously described techniques [30]. Briefly, mesenteric arteries were rapidly dissected and frozen in order to maintain their *in vivo* activity (phosphorylated targets). Proteins were extracted and the total protein content was determined by the Bradford technique [31] in order to file equal amounts (15μg) of the denatured proteins. Membranes were incubated with polyclonal antibodies directed against MLC, phosphorylated MLC (pMLC), filamin-1, profilin-1, P38 MAP kinase (P38), phosphorylated P38 (pP38), protein kinase A (PKA), phosphorylated PKA (pPKA), RhoA and IP receptor. The detection was performed by chemiluminescence emitted from luminol oxidized by peroxidase (ECL system, Amersham). Each protein expression was compared to β-actin.

### RhoA-GTP measurement

RhoA-GTP was measured in mesenteric artery segments using a commercially available kit (G-LISA® RhoA Activation Assay Biochem Kit^TM^, BK124, Cytoskeleton Inc.).

### Statistical analysis

Results are expressed as means ± SEM. Significance of the differences between groups was determined by analysis of variance (2-way ANOVA for concentration-response curves) or a non-parametric Mann-Whitney test. Values of p<0.05 were considered to be significant. All statistical analysis was performed using Graphpad Prism® software.

## Results

### Effect of maternal hyperglycaemia on body weight, glycaemia and arterial blood pressure in offspring

Hyperglycaemia of mother rats ranged from 15 to 25mmol/l induced normal gestational and delivery conditions as previously described [32]. For rats delivered spontaneously at term (21 days); the number of pups per litter was similar in both CMO and DMO groups (13.3 ± 0.7 and 11.1 ± 0.9 pups, for CMO and DMO respectively) with an equivalent birth weight (10.42 ± 0.20grs for CMO and 10.41 ± 0.40grs for DMO). As shown in Table 1, body weight was lower in 18 month-old DMO than in age-matched CMO (Table 1). Nevertheless, no difference in glycaemia was found between the 2 groups. Finally, mean arterial blood pressure (MBP) was significantly higher in 18 month-old DMO, compared to aged-matched CMO and 3 month-old DMO (Table 1).

**Table 1:** Physiological parameters of control mother offspring (CMO) and diabetic mother offspring (DMO).

	3 months		18 months	
	*CMO*	*DMO*	*CMO*	*DMO*
**Body weight (g)**	385.6 ± 20.8	376.3 ± 13.2	797.5 ± 52.7	626.0 ± 18.5*
** **				
**Glycaemia (mmol/l)**	7.7 ± 0.5	6.8 ± 0.4	10.3 ± 1.8	13.9 ± 3.2
				
**MBP (mmHg)**	93.5 ± 3.9	99.2 ± 4.0	98.4 ± 7.8	125.3 ± 4.3*
				

At 3 months of age, n = 16 CMO and 12 DMO for body weight and glycaemia, n = 7 CMO and 7 DMO for mean blood pressure (MBP); at 18 months of age, n = 11 CMO and 11 DMO for body weight and glycaemia, n = 6 CMO and 4 DMO for blood pressure. Values are mean ± SEM.

* p<0.05 DMO vs. CMO.

### Isolated mesenteric arteries relaxation in offspring

**Fig 1:**
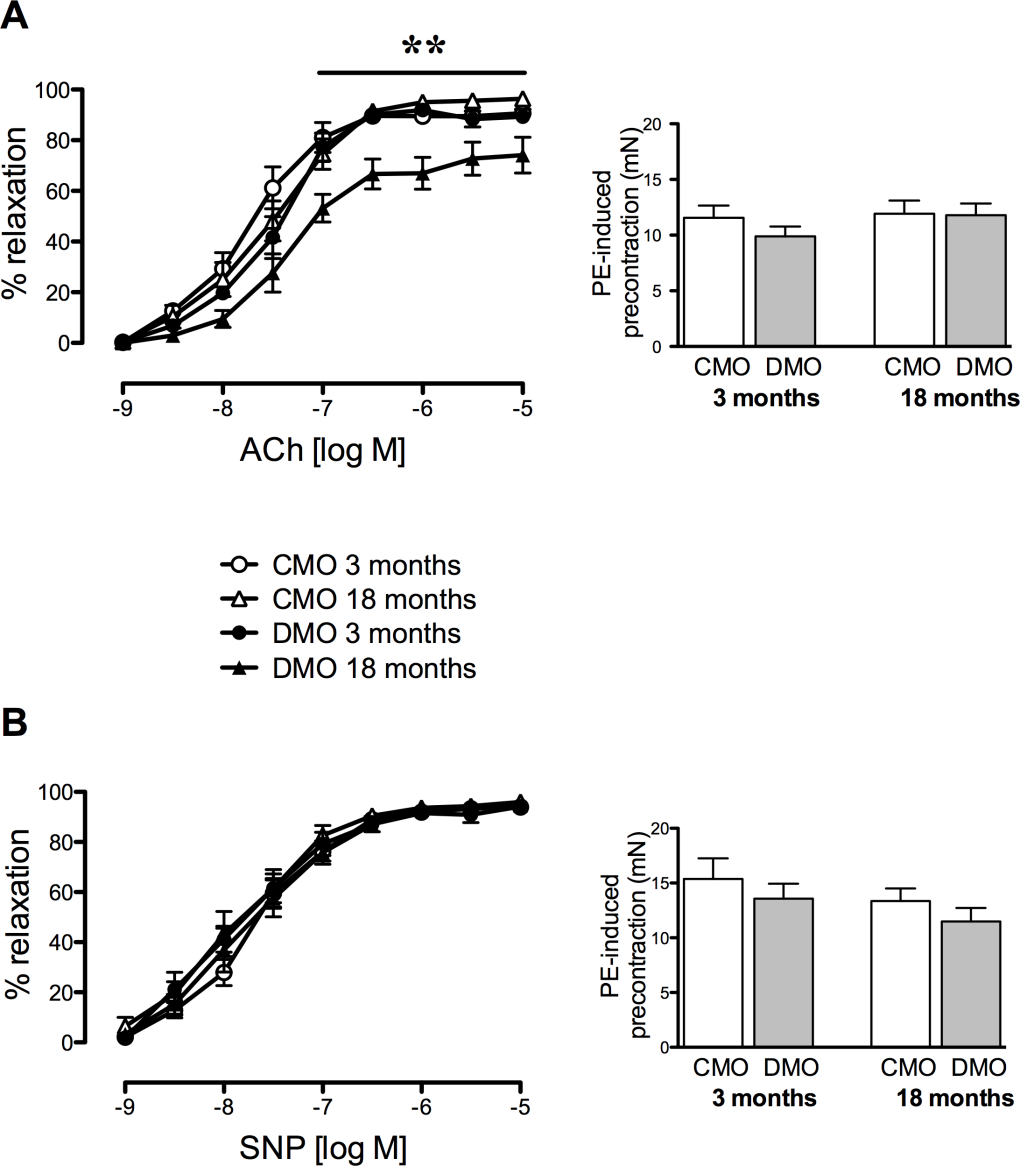
Vascular relaxation of mesenteric arteries in control (CMO) and diabetic (DMO) mother offspring. Concentration-response curve to acetylcholine (ACh, A) and sodium nitroprusside (SNP, B). Bar graphs represent the level of pre-contraction induced by 10^-6^M phenylephrine. Values are mean ± SEM (n = 7 minimum per group). * p<0.05 and ** p<0.01 DMO vs. CMO.

Acetylcholine induced a concentration-dependent relaxation of isolated mesenteric resistance arteries. Achetycholine-mediated relaxation was significantly lower in mesenteric arteries of 18-month old DMO, compared to age-matched CMO and compared to 3-month old DMO. However, PE-induced pre-contraction was equivalent (Fig 1A). Endothelium-independent (SNP) relaxation was not affected in DMO (Fig 1B).

### Effect of maternal hyperglycaemia on the prostacyclin pathway in offspring

**Fig 2:**
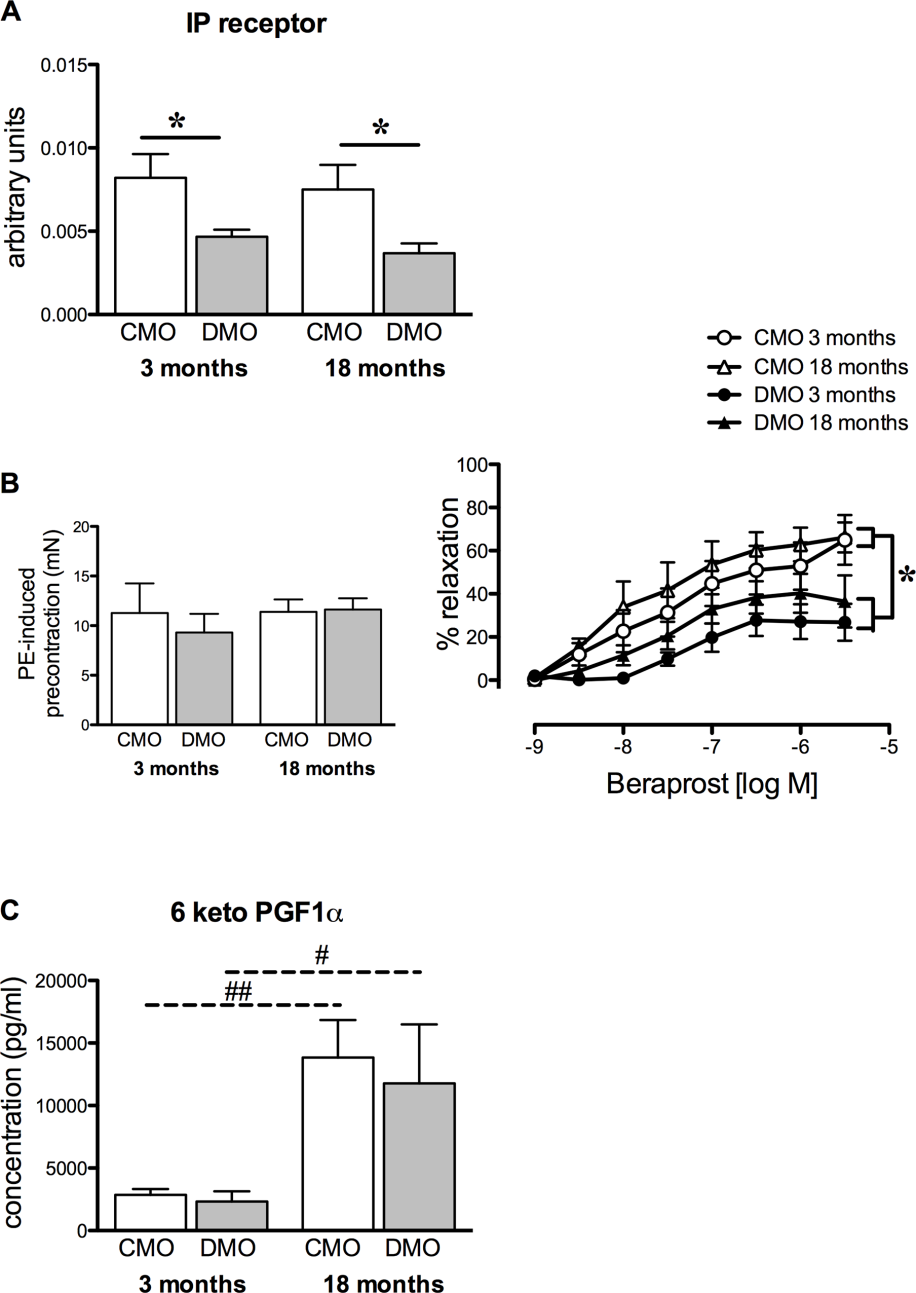
Prostacyclin-mediated relaxation and receptor expression level in control (CMO) and diabetic (DMO) mother offspring. (A) Relative protein expression of the prostacyclin receptor (IP receptor) was analysed by Western-blot in mesenteric arteries of CMO (open bars, n = 5) and DMO (solid bars, n = 5); each value was normalised to β-actin protein expression. (B) Left panel represents phenylephrine (PE)-induced pre-contraction level; right panel shows concentration-relaxation response curve to beraprost of mesenteric arteries in CMO and DMO (n = 5 at 3 months and n = 7 at 18 months of age for each group). Values are mean ± SEM. (C) Measurement of serum 6-keto-PGF1-α (prostacyclin metabolite) in CMO (n = 6) and DMO (n = 6). Each bar graph represents mean ± SEM. * p<0.05 DMO vs. CMO and ## p<0.01 18 vs. 3 months-old animals.

In both 3- and 18-month old DMO, the IP receptor expression level in mesenteric resistance arteries was significantly decreased compared to age-matched CMO (Fig 2A). This was associated with a significant reduction in beraprost-induced relaxation, 3- and 18-month old DMO compared to age-matched CMO (Fig 2B). The level of 6-keto-PGF1α increased with age and was equivalent in DMO and CMO.

### Vasoconstriction pathways of mesenteric arteries in offsprings

**Fig 3:**
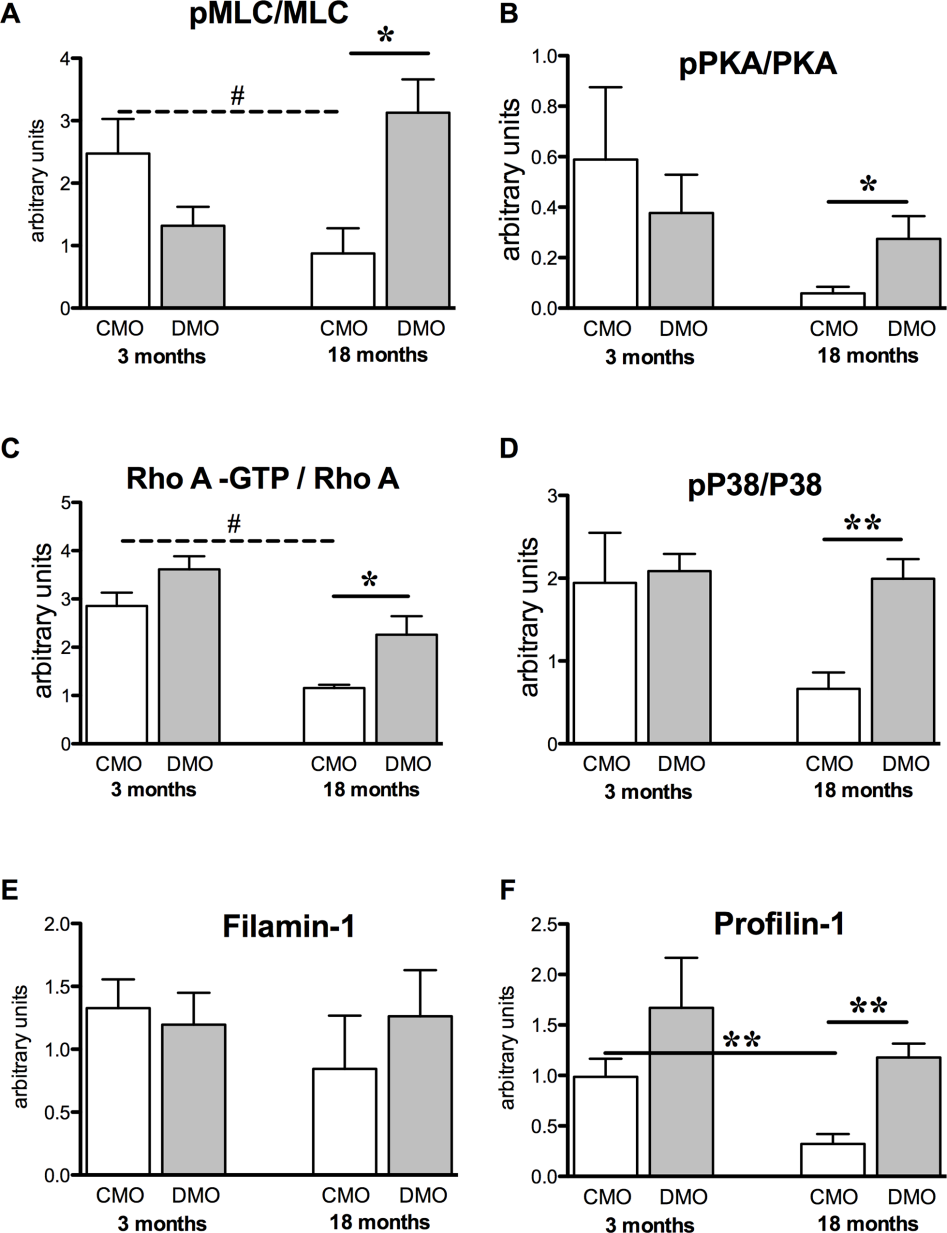
Western blot analysis of contractile proteins. (A) Activity of MLC reflected by the pMLC/MLC ratio; (B) activity of PKA reflected by the pPKA/PKA ratio; (C) activity of RhoA reflected by the RhoA-GTP/RhoA ratio; (D) activity of MAP kinase P38 reflected by the ratio of pP38/P38 and protein expression levels of (E) filamin-1 and (F) profilin-1, normalised to β-actin were measured in mesenteric arteries from 3- and 18-month old CMO and DMO. Values are mean ± SEM (n = 5 minimum per group). * p<0.05 and ** p<0.01 DMO vs. CMO at the same age, ## p<0.05 18 vs. 3 months of age.

Whereas MLC protein level was similar in CMO and DMO at 3 and 18 months of age (S1 Fig), we observed a significant decrease in pMLC protein expression level in DMO at 3 months, and an increase at 18 months (S1 Fig). Thus in DMO, the pMLC/MLC ratio was slightly decreased at 3 months but 3-fold increased at 18 months of age (Fig 3A). This was associated with an increased pPKA/PKA ratio (Fig 3B and S1 Fig) and activated RhoA protein (Fig 3C and S1 Fig). We analysed MAPK P38 activity and found a similar expression level of P38 in CMO and DMO (S2 Fig), whereas the activity reflected by the ratio of phospho-P38/P38 was significantly increased in DMO at 18 months of age (Fig 3D). Finally, we observed that filamin-1 protein expression, which is implicated in actin reticulation, was similar between CMO and DMO both at 3 and 18 months (Fig 3E), whereas profilin-1, which supports actin polymerisation, significantly increased in 18-month-old DMO (Fig 3F).

### Contractility of isolated mesenteric arteries and histomorphology in offsprings

**Fig 4:**
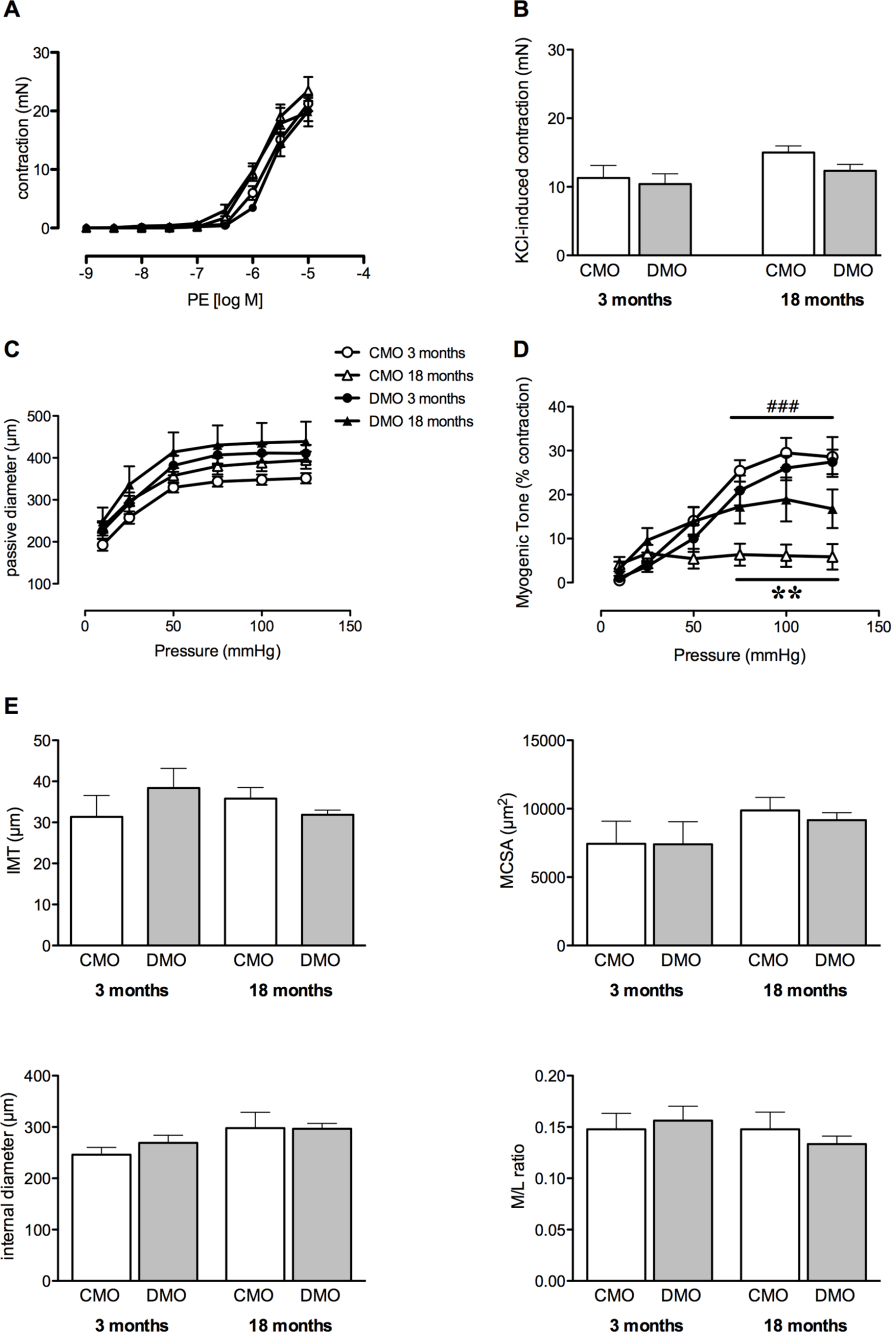
Vascular contraction and histomorphological parameters of mesenteric arteries from control (CMO) and diabetic (DMO) mother offspring. (A) Concentration–response curve to phenylephrine (PE); (B) KCl-induced contraction; (C) passive arterial diameter measured in response to increased pressure; (D) myogenic tone and (E) lumen diameter, media cross-sectional area (MCSA), intima-media thickness (IMT) and remodelling index (media/lumen ratio). Values are mean ± SEM (n = 5 minimum per group). ** p<0.01 DMO vs. CMO at 18 months of age; ### p<0.001 CMO 18 vs. CMO 3.

Phenylephrine- and KCl-mediated contraction were similar in 3- and 18-month-old DMO and CMO (Fig 4A and 4B).

Stepwise increases in pressure induced myogenic tone in third order mesenteric resistance arteries. Passive arterial diameter was not significantly different between groups (Fig 4C). Interestingly, in 18-month old DMO, myogenic tone was maintained at a high level, equivalent to that of 3-month old DMO, although myogenic tone decreased with age in CMO (Fig 4D).

Lumen diameter, media cross sectional area (MCSA) and intima media thickness (IMT) were were not significantly different between CMO and DMO and not affected by age (Fig 4E). In addition, the media/lumen ratio was not significantly different in DMO and CMO (Fig 4E).

## Discussion

Resistance arteries play a key role in the control of peripheral vascular resistance, tissue perfusion and, as a result, in cardiovascular homeostasis [33]. As resistance arteries are the main determinant of tissue blood flow and subsequently blood pressure, their vascular reactivity was analysed. Our study evidenced modifications of mesenteric artery vasodilation function in rats exposed to maternal hyperglycaemia at as early as 3 months, before the development of hypertension. Indeed, we found an early decreased IP receptor protein level which had a functional repercussion on vascular relaxation, which worsened thereafter with endothelial dysfunction and an elevated myogenic tone. Although endothelial dysfunction (i.e. alteration in the NO pathway) has been described in numerous experimental models of fetal programming [19,20,34] and detected in our model (decreased PeNOS/eNOS ratio in DMO, data not shown), sustainable changes in the vascular PGI2/IP pathway and late perturbation in myogenic tone have never been reported, to the best of our knowledge.

In 3-month old DMO rats, we identified an early alteration in PGI_2_-induced vasodilator response of resistance arteries. We demonstrated that this reduction in PGI_2_-dependent relaxation was associated to a decrease in IP receptor expression level. These results, obtained in resistance arteries, are in agreement with the assumption that modifications affecting the IP receptor could contribute to hypertension in later life [25]. The PGI_2_/IP receptor-mediated vasodilation involves the cAMP pathway and might be implicated in BP regulation [35]. Previous studies have demonstrated that genetic inactivation of PGI_2_ synthase causes significant alterations in the architecture of the vascular wall, including thickening of the arterial media and consequently, an increase in arterial BP [36]. Nevertheless, the effector appeared to be the IP receptor instead of the level of PGI_2_. Indeed, decreased IP receptor levels was not associated with changes in PGI_2_ generation as evidenced by the absence of modification in serum 6-keto-PGF1α in DMO compared to CMO. This is in agreement with a previous study showing no change in urine and plasma 6-keto-PGF1α levels in mice lacking the IP receptor [37]. The high 6-keto-PGF1α concentration observed in 18 month-old rats could be a consequence of an age-related increase in vascular COX-2 mRNA and protein expressions as previously demonstrated in the rat aorta [38,39]. Although studies involving mice lacking the IP receptor show contradictory results regarding baseline blood pressure, due to differences in the mice’s genetic background [35,37,40], during salt loading, the IP receptor deficiency led to more severe hypertension in comparison to wild-type mice [35,40].

Later in life, DMO developed a deeper endothelial dysfunction besides altered beraprost-induced vasodilation. Indeed, in 18-month-old DMO, endothelium (acetylcholine)-dependent relaxation decreased, even though endothelium-independent (SNP)-induced vasodilation remained unchanged. The diminished endothelium-dependent relaxation in 18 month old DMO could be correlated to a higher myogenic tone of the mesenteric arteries. Thus, this decreased activity associated with the diminished effect of PGI_2_, results in unbalanced vasoconstriction functions followed by increased blood pressure.

In DMO rats, vasoconstriction induced by PE or KCl was not affected, suggesting that the contractile apparatus was preserved. In agreement, restricted or low-protein diets of mothers during pregnancy have shown similar results [41,42], suggesting that fetal programming of cardiovascular disorders could not be due to alteration of the contractile apparatus. Despite these observations, we showed that pressure-induced MT was maintained at a high level in 18-month old DMO compared to age-matched CMO. However, MT has been shown to decrease with age in mice or rats [43,44]. The increased MT may be related to the high blood pressure found in 18 month-old DMO since elevated MT has been reported during the early stages of hypertension development in mouse and rat models [44,45,46]. Pressure or stretch applied to smooth muscle cells induces changes cytoskeletal organization, including actin polymerization and phosphorylation of MLC [11], due to RhoA activation [47]. In 18-month old DMO, we observed higher pMLC/MLC and pPKA/PKA ratios and greater RhoA activity, compared to age-matched CMO and to the 3-month old rats. In addition, we also found an increase in profilin-1 expression levels in 18-month old DMO; this latter finding is in agreement with recent studies showing that profilin-1 is involved in MT [16,48]. However, in contrast to another study showing that filamin-1 is also involved in MT [49], filamin-1 was not affected. Similarly, we observed the maintenance of a high level of pP38/P38 ratio in mesenteric arteries of 18-month old DMO, even though several studies have shown a weak involvement of the P38 MAP kinase pathway in myogenic tone [47,50,51]. Nevertheless, we may hypothesize that some minor pathways such as the P38 MAP kinase pathway might play a more important role in MT in old DMO thus maintaining an elevated level of pressure-induced vasoconstriction. We may also postulate that the high level of MT found in 18-month old DMO results in the maintenance of RhoA expression levels, as RhoA is essential for MT [47]. The high PKA activity observed in arteries of 18-month old DMO could explain this high RhoA level, as PKA activity modulates MT [52], and this could involve a PKA-dependent Ser188 phosphorylation, which protect RhoA from degradation [53].

## Conclusion

Our study shows that exposure to maternal hyperglycaemia involves early vascular perturbations in relation to reprogramming of the PGI_2_/IP pathway. The decreased IP expression is constant over time, although its effect seems to alter vascular response in an age-dependent manner. Second, changes in vascular function are in favour of vasoconstriction, which could take part to the hypertension observed in adult DMO. Thus, the decreased arterial IP expression, which does not affect vascular function in 3 month-old rats, could amplify the vascular dysfunction associated.

In humans, a natural mutation of the IP receptor (IP^R212C^) is associated with impaired PGI2/IP pathway leading to atherothrombosis and coronary heart disease [54,55]. A more recent study has described a dominant negative action of IP^R212C^ on wild-type IP function, which enhances endoplasmic reticulum retention of the IP receptor and reduces signalling in response to a PGI_2_ analogue [56].

Thus, perinatal resetting in the PGI2/IP pathway seems to be significant and precocious of the development of cardiovascular disease. A better understanding of mechanisms implicated in this resetting of vascular functions by maternal hyperglycaemia would certainly open new epidemiologic and therapeutic perspectives.

## Supporting Information

S1 Fig Protein expression levels of (A) MLC, (B) phosphorylated-MLC, (C) PKA, (D) phosphorylated PKA, (E) RhoA normalized to β-actin and (F) RhoA activity in mesenteric arteries from CMO and DMO rats at 3 and 18 month of age. Open bars represent CMO values and solid bars represent DMO values. Values are mean ± SEM (n = 5 minimum per group).
(TIF)

S2 Fig Protein expression levels of (A) P38 and (B) phosphorylated-P38 (C) normalized to β-actin in mesenteric arteries from CMO and DMO rats at 3 and 18 month of age. Open bars represent CMO values and solid bars represent DMO values. Values are mean ± SEM (n = 5 minimum per group).
(TIF)

S3 Fig Western blot analysis of mesenteric arteries of CMO and DMO at 3 months of age. The following proteins were analysed: prostacyclin receptor (IP Rc), beta-actin (β actin), RhoA, profilin-1 (profilin), filamin-1 (filamin), P38 MAP kinase (P38), phosporylated P38 MAP kinase (pP38), myosin light chain (MLC), phosphorylated myosin light chain (pMLC), protein kinase A (PKA) and phosphorylated protein kinase A (pPKA).
(TIF)

S4 Fig Western blot analysis of mesenteric arteries of CMO and DMO at 18 months of age. The following proteins were analysed: prostacyclin receptor (IP Rc), beta-actin (β actin), RhoA, profilin-1 (profilin), filamin-1 (filamin), P38 MAP kinase (P38), phosporylated P38 MAP kinase (pP38), myosin light chain (MLC), phosphorylated myosin light chain (pMLC), protein kinase A (PKA) and phosphorylated protein kinase A (pPKA).
(TIF)
