# Supplementary figures and images for: Long Lasting Microvascular Tone Alteration in Rat Offspring Exposed In Utero to Maternal Hyperglycaemia
Source: PLoS One. 2016 Jan 12;11(1):e0146830. doi: 10.1371/journal.pone.0146830 (PMC4710502; doi:10.1371/journal.pone.0146830)

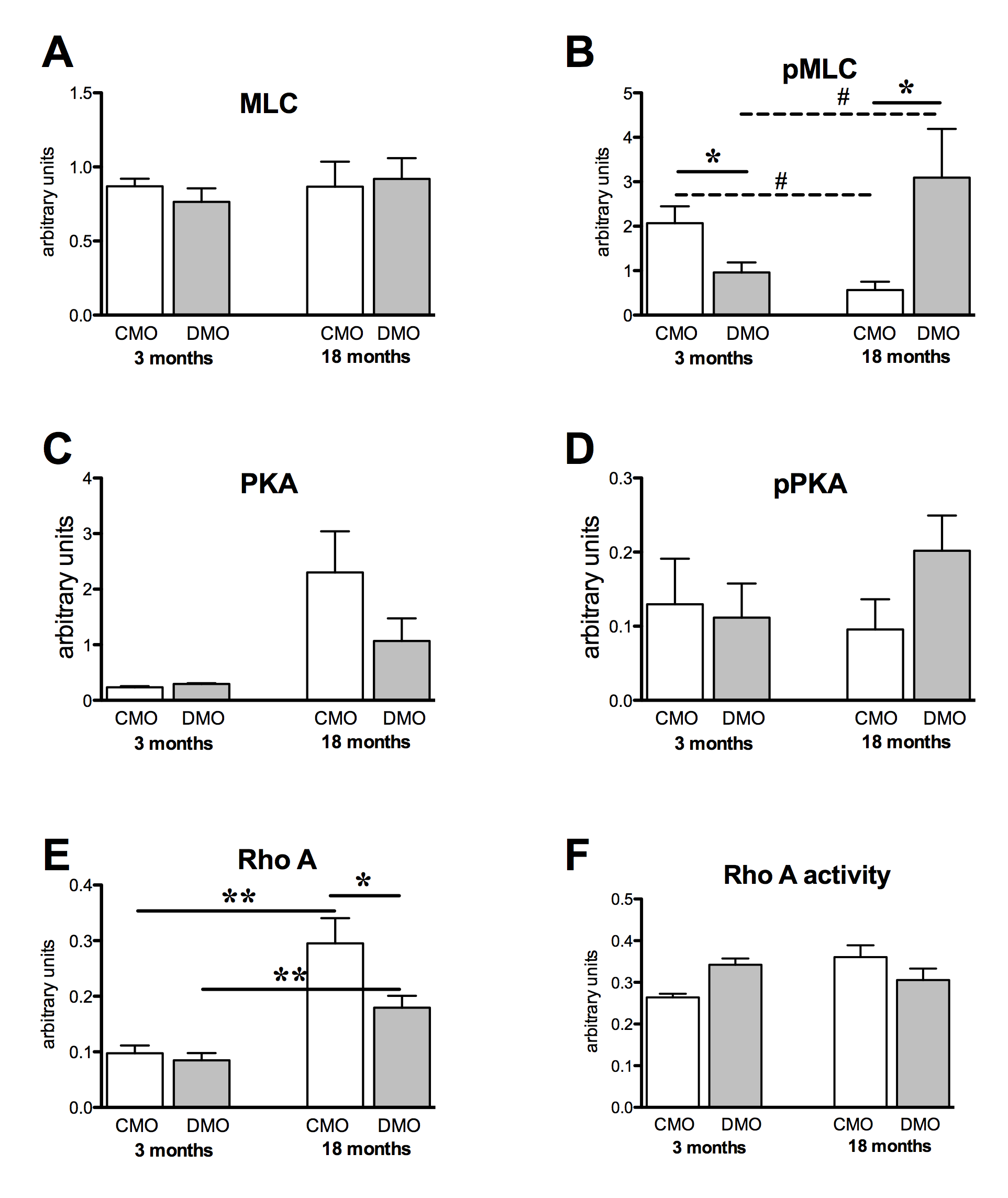

Supplement: S1 Fig — Open bars represent CMO values and solid bars represent DMO values. Values are mean ± SEM (n = 5 minimum per group). (TIF) [file pone.0146830.s001.tif]

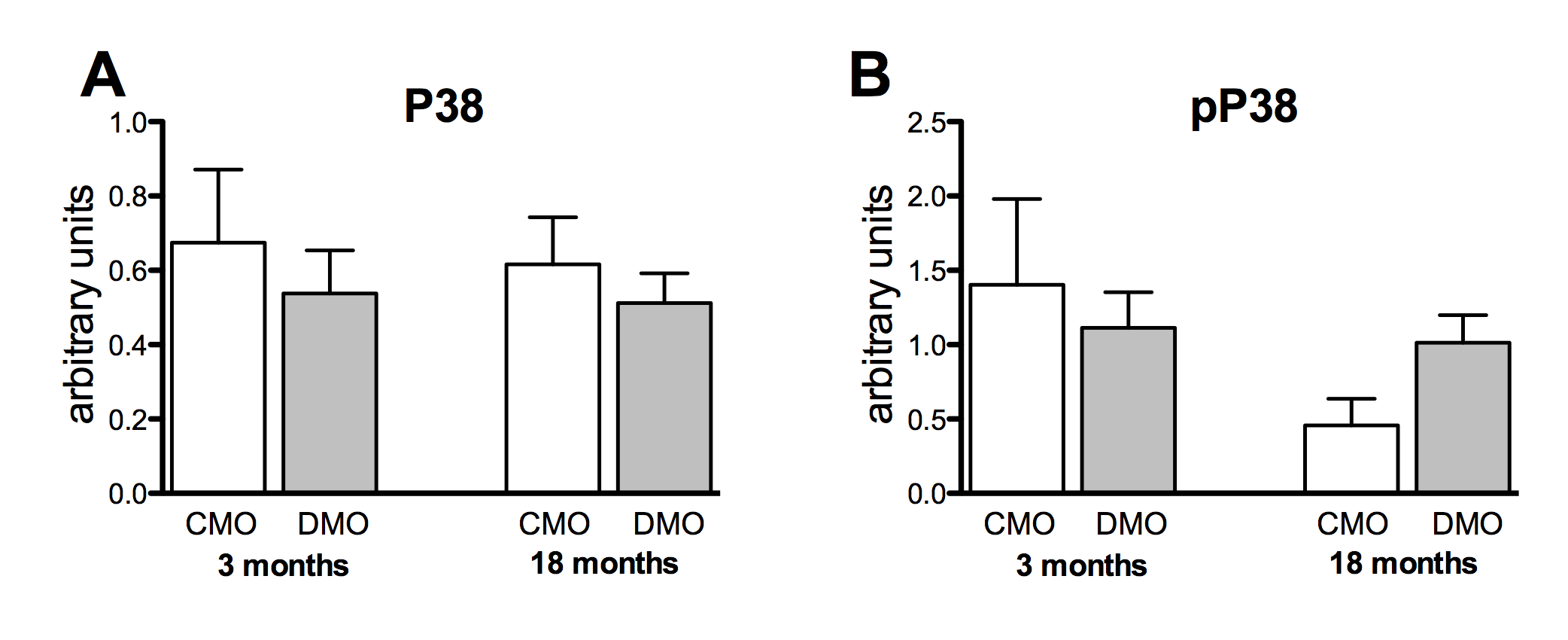

Supplement: S2 Fig — Open bars represent CMO values and solid bars represent DMO values. Values are mean ± SEM (n = 5 minimum per group). (TIF) [file pone.0146830.s002.tif]

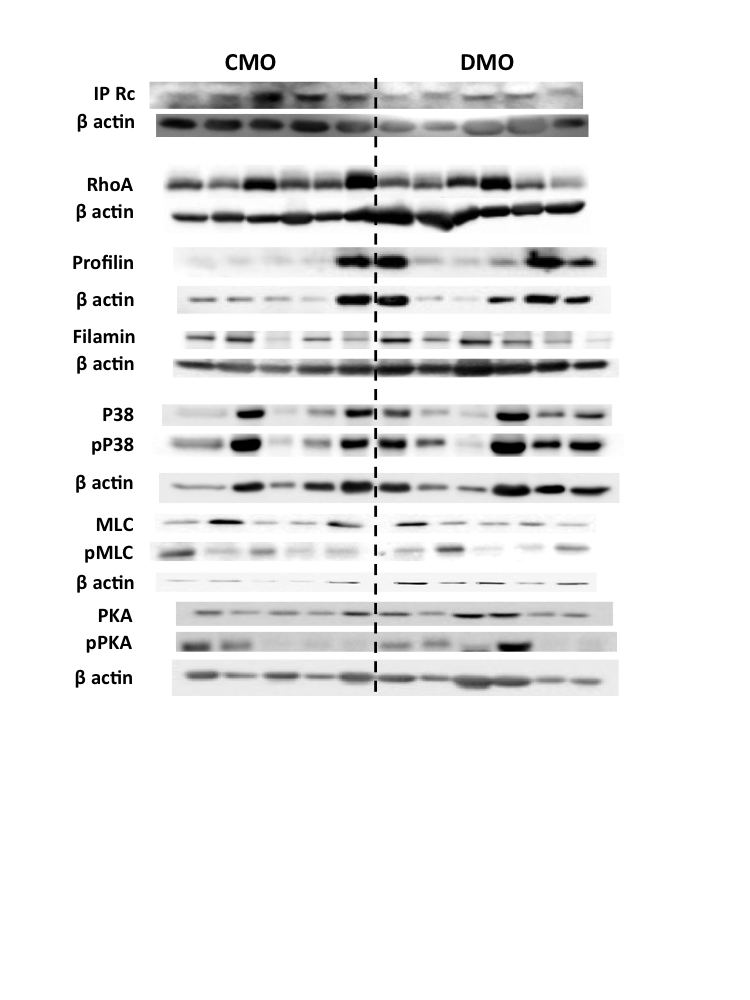

Supplement: S3 Fig — The following proteins were analysed: prostacyclin receptor (IP Rc), beta-actin (β actin), RhoA, profilin-1 (profilin), filamin-1 (filamin), P38 MAP kinase (P38), phosporylated P38 MAP kinase (pP38), myosin light chain (MLC), phosphorylated myosin light chain (pMLC), protein kinase A (PKA) and phosphorylated protein kinase A (pPKA). (TIF) [file pone.0146830.s003.tif]

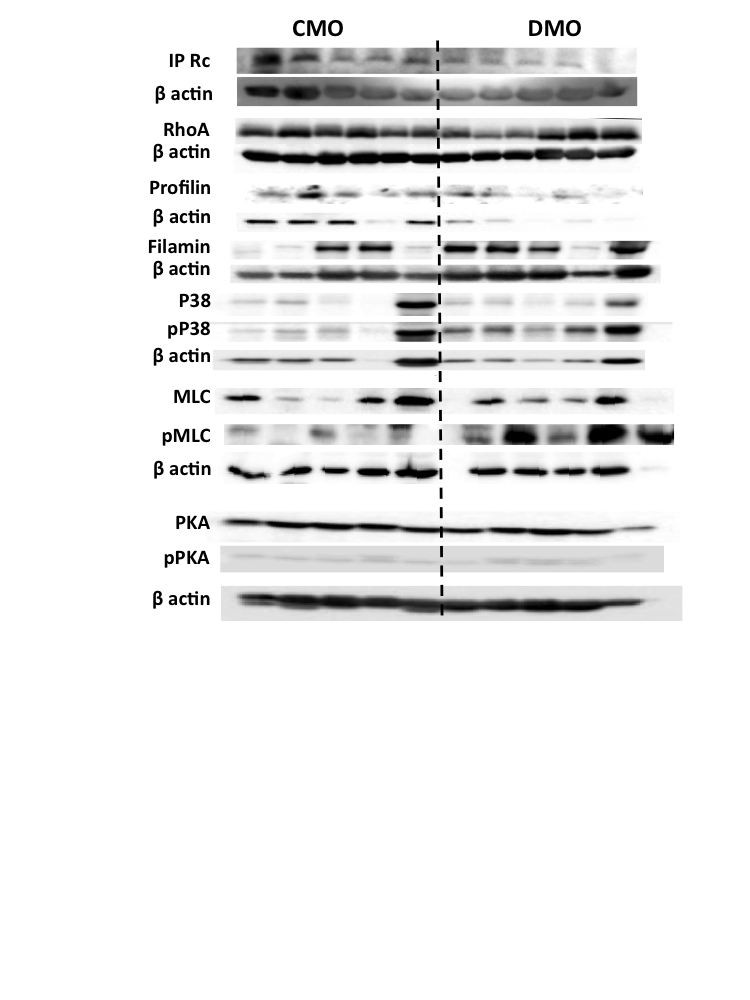

Supplement: S4 Fig — The following proteins were analysed: prostacyclin receptor (IP Rc), beta-actin (β actin), RhoA, profilin-1 (profilin), filamin-1 (filamin), P38 MAP kinase (P38), phosporylated P38 MAP kinase (pP38), myosin light chain (MLC), phosphorylated myosin light chain (pMLC), protein kinase A (PKA) and phosphorylated protein kinase A (pPKA). (TIF) [file pone.0146830.s004.tif]
